# G protein-coupled receptors: Role in metabolic disorders

**DOI:** 10.3389/fendo.2022.984253

**Published:** 2022-08-04

**Authors:** Luiz F. Barella, Shanu Jain, Sai P. Pydi

**Affiliations:** ^1^ Lilly Diabetes Center of Excellence, Indiana Biosciences Research Institute, Indianapolis, IN, United States; ^2^ Molecular Recognition Section, Laboratory of Bioorganic Chemistry, National Institute of Diabetes and Digestive and Kidney Diseases, National Institutes of Health, Bethesda, MD, United States; ^3^ Department of Biological Sciences and Bioengineering, Indian Institute of Technology, Kanpur, India

**Keywords:** GPCR (G protein-coupled receptor), type 2 diabetes (T2D), metabolic disease, β-arrestin, obesity

G protein-coupled receptors (GPCRs) are seven-transmembrane proteins that are essentially expressed on every cell type in the body. Many physiological and pathological processes are controlled by GPCRs. When an agonist binds to a GPCR, it activates G proteins, which in turn stimulate a slew of secondary messengers (1). It has been shown that β-arrestins, a class of intracellular proteins that regulate GPCR activity, can also act as signaling molecules, opening up a new world of signaling possibilities. Drugs targeting GPCRs account for more than a third of all currently accessible commercially available medications (2), making them an attractive therapeutic option for the treatment of obesity and related metabolic diseases, including type 2 diabetes (T2D). In the current Research Topic, we compiled various articles that exemplify the significance of GPCR signaling in metabolic diseases, which reiterates their potential as therapeutic targets for the treatment of several disorders.

To indicate the significance of GPCR signaling pathways in obesity and T2D, Souza et.al. reviewed the role of GPR35, GPR40, GPR41, GPR43, GPR81, GPR119, and GPR120 in the regulation of insulin secretion, insulin sensitization, β-cell expansion, and glucose homeostasis. Further, the author emphasizes the importance of β-arrestin, which can exhibit G protein independent signaling in adipocytes, β-cells, agouti-related peptide neurons, hepatocytes, and skeletal muscle. The review further reiterates the importance of developing G-protein or β-arrestin biased drugs that can activate specific signaling pathways for the therapy of metabolic disorders and evade the activation of side-effects causing or undesired signaling pathways.

Extracellular nucleotides and nucleotide sugars serve as signaling molecules for cell surface purinergic receptors (3). Purinergic receptors are found in a variety of liver resident cell types and regulate hepatic metabolism in health and disease. Jain and Jacobson provide a comprehensive insight into the developing role of two families of purinergic receptors- adenosine (P1) and P2 receptors in the pathogenesis of the non-alcoholic fatty liver disease (NAFLD), steatohepatitis, liver fibrosis, and liver cancer. The authors highlighted that pre-clinical studies targeting A_3_, A_2A_, A_2B_, P2Y_6_, and P2Y_14_ receptors show promise for treating liver metabolic disorders. Numerous potent ligands exist for purinergic receptors (4), which can be tested in hepatic cell-specific knockout mouse models to advance drug discovery for liver metabolic disorders.

The severity of liver fibrosis is predictive of liver-related mortality and morbidity in individuals with NAFLD (5). A riveting review by Kimura et al reinforces the relevance of hepatic stellate cells (HSC) GPCRs in the pathogenesis of liver fibrosis. HSCs are primary cells that on activation secrete extracellular matrix proteins resulting in liver fibrosis. The authors describe the role of Gs, Gq, Gi, and G12/13 G protein-coupled receptors in regulating HSC biological processes including cell proliferation, differentiation, activation of myofibroblasts, and collagen production. Interestingly, most of the HSCs GPCRs enhance liver fibrosis, while few are involved in the suppression of fibrosis. Understanding the role of GPCRs expressed in HSC may lead to the development of novel drugs for the treatment of fibrosis and NAFLD.

Ghrelin is the gut-derived peptide hormone with an orexigenic effect (6). An intriguing review by Khelifa et al discusses the connection between ghrelin receptor signaling and anorexia nervosa-a complex disease characterized by metabolic and psychological dysfunction. Since ghrelin increases food intake, improves gut motility, and increases the pleasure and motivation related to food intake, ghrelin receptor agonist could be a potential pharmacological target for treating anorexia nervosa. Moreover, ghrelin signaling pathways exhibit close anatomical and functional connectivity with dopaminergic pathways, forming a receptor couple in anorexia nervosa. The authors emphasize the pleiotropic effects of ghrelin receptor signaling through multiple G proteins and β-arrestin pathways which mandate the development of biased agonists to prevent the adverse effects of the drugs. Drug discovery must also consider the close connection between these receptor coupling properties to result in the effective treatment of anorexia nervosa.

Metformin is an approved antidiabetic agent used in patients with T2D (7). Herein, Franco et al. made interesting observations regarding the impact of metformin on the autonomic nervous system (ANS) and metabolism in a rat model of obesity. In this study, the authors observed augmented activity of the vagus nerve, hyperinsulinemia, and hyperglycemia in obese rats. Interestingly, chronic treatment of metformin led to a decrease in vagal activity. Also, in obese animals, the response of the muscarinic type 3 GPCRs was increased in pancreatic islets, which associates with the elevated insulin secretion response and insulin plasma levels observed. They concluded that chronic metformin ameliorates the deficits of obesity along with the normalization of ANS activity.

GLP-1 and GIP are well-known incretin hormones capable of augmenting glucose-stimulated insulin production and thereby contributing to glucose clearance from the circulation (8). Ovlund et al. explored the potential contribution of these incretin receptors to insulin-independent processes for glucose-lowering. They studied glucose elimination rates in wild-type mice and mice with the deletion of GLP-1 or GIP receptors. To exclude the involvement of insulin in glucose clearance, they employed diazoxide. Interestingly, their study showed that glucose clearance is decreased in mice lacking GLP-1 receptors, suggesting that active GLP-1 receptors are necessary for insulin-independent glucose clearance. Conversely, GIP receptors seem to be dispensable in this process. This study showed a dissociated effect of incretin hormone receptors on insulin-independent glucose disposal in mice, in which GLP-1 receptors seem to have a greater impact.

Next, Miller et al. reviewed the roles of cholecystokinin (CCK) in the maintenance of nutritional homeostasis and its potential role in the treatment of obesity. CCK is a peptide known for its important role in the regulation of nutritional homeostasis and is secreted by the I cells in the gut (9). Focusing on satiety control, the authors gathered the historical aspects of CCK’s discovery as well as the physiology of its receptors. The authors reviewed the challenge that it has been to develop clinical candidates to treat obesity since the results of clinical trials have disappointed, mostly due to adverse effects such as nausea and diarrhea. Finally, they explore the development of biased and allosteric modulators as potential strategies for new drugs targeting CCK receptors, which could circumvent the undesirable side effects observed with the current compounds tested to the day.

Wess assesses the significance of Designer GPCRs (a.k.a. DREADDs) as chemogenetic tools to investigate signaling cascades. In this opinion article, Wess reviews the mode of action of DREADDs and explores the advantages of using them for experiments, particularly *in vivo*. The author also briefly summarized previously published studies that explored the impact of expressing and activating DREADDs in different mouse tissues/cell types, in the context of obesity and diabetes. Wess also reaffirms the current challenges that remain even after the promising discoveries enabled by the study with DREADDs and expects that the insightful information gained by these studies will lead to the development of new drugs for the treatment of metabolic diseases.

Finally, we would like to thank the reviewers for their meticulous evaluation of the articles and the authors for their contribution to the special issue. Hopefully, this Research Topic will provide comprehensive knowledge on the role of GPCR signaling in various metabolic tissues and contribute to our understanding of GPCRs in metabolic disorders.

## Author contributions

All authors listed have made a substantial, direct, and intellectual contribution to the work and approved it for publication. All authors contributed equally to the writing of this editorial.

## Funding

SPP laboratory is supported by SERB – Core Research Grant (CRG/2021/004502), DBT Wellcome Trust India Alliance (IA/I/21/1/505613) and start up grant from IIT-Kanpur.

## Conflict of interest

The authors declare that the research was conducted in the absence of any commercial or financial relationships that could be construed as a potential conflict of interest.

## Publisher’s note

All claims expressed in this article are solely those of the authors and do not necessarily represent those of their affiliated organizations, or those of the publisher, the editors and the reviewers. Any product that may be evaluated in this article, or claim that may be made by its manufacturer, is not guaranteed or endorsed by the publisher.
